# Epstein–Barr Virus Latent Membrane Protein 2A (LMP2A) Enhances ATP Production in B Cell Tumors through mTOR and HIF-1α

**DOI:** 10.3390/ijms25073944

**Published:** 2024-04-02

**Authors:** Ryan Incrocci, Rosalinda Monroy Del Toro, Grace Devitt, Melody Salimian, Kamaljit Braich, Michelle Swanson-Mungerson

**Affiliations:** 1 Department of Microbiology and Immunology, College of Graduate Studies, Midwestern University, Downers Grove, IL 60515, USA; 2 Chicago College of Osteopathic Medicine, Midwestern University, Downers Grove, IL 60515, USA; grace.devitt@midwestern.edu (G.D.); melody.salimian@midwestern.edu (M.S.);

**Keywords:** Epstein–Barr Virus, metabolism, latency membrane protein 2A (LMP2A), mammalian target of rapamycin (mTOR)

## Abstract

Epstein–Barr Virus (EBV) exists in a latent state in 90% of the world’s population and is linked to numerous cancers, such as Burkitt’s Lymphoma, Hodgkin’s, and non-Hodgkin’s Lymphoma. One EBV latency protein, latency membrane protein 2A (LMP2A), is expressed in multiple latency phenotypes. LMP2A signaling has been extensively studied and one target of LMP2A is the mammalian target of rapamycin (mTOR). Since mTOR has been linked to reprogramming tumor metabolism and increasing levels of hypoxia-inducible factor 1 α (HIF-1α), we hypothesized that LMP2A would increase HIF-1α levels to enhance ATP generation in B lymphoma cell lines. Our data indicate that LMP2A increases ATP generation in multiple Burkitt lymphoma cell lines that were dependent on HIF-1α. Subsequent studies indicate that the addition of the mTOR inhibitor, rapamycin, blocked the LMP2A-dependent increase in HIF-1α. Further studies demonstrate that LMP2A does not increase HIF-1α levels by increasing HIF-1α RNA or STAT3 activation. In contrast, LMP2A and mTOR-dependent increase in HIF-1α required mTOR-dependent phosphorylation of p70 S6 Kinase and 4E-BP1. These findings implicate the importance of LMP2A in promoting B cell lymphoma survival by increasing ATP generation and identifying potential pharmaceutical targets to treat EBV-associated tumors.

## 1. Introduction

Epstein–Barr Virus (EBV) is a ubiquitous human herpes virus that infects B lymphocytes in 90% of the world’s population [1]. While a benign viral latency persists in most individuals, it can also manifest in human disease years after primary infection as EBV-associated cancers including Burkitt’s Lymphoma, Hodgkin’s Lymphoma, non-Hodgkin’s lymphomas of AIDS, nasopharyngeal carcinoma and gastric carcinoma [2,3,4,5].

While the initial infection of EBV is often asymptomatic or results in infectious mononucleosis, the latent viral state after initial infection serves as a reservoir for the precursors of malignant B cell transformation in EBV-associated lymphomas [6,7]. There are multiple stages of EBV latency, which are defined by differences in gene expression [8]. However, one EBV latency protein, Latent membrane protein 2A (LMP2A), is identified in both fully developed EBV lymphomas and latently-infected B cells, which serve as the precursor to malignant EBV lymphomas [9].

The importance of LMP2A signaling for the survival of EBV latently-infected B cells is well established [9]. LMP2A mimics the upstream signals from the B cell receptor signaling [10,11] due to the presence of an immunoreceptor activation motif (ITAM) in its long cytoplasmic amino-terminal domain [12]. This motif is responsible for binding and activating Syk kinase that subsequently activates downstream pathways that promote both proliferation and survival. In newly infected cells, LMP2A is important for augmenting the initial proliferation induced by EBV infection that is required for EBV transformation in vitro [13]. Furthermore, in a mouse model of Burkitt’s lymphoma, LMP2A enhanced MYC-driven cell cycle progression by increasing the protein instability of a the tumor suppressor p27^kip1^ [14]. Additionally, LMP2A signaling induces the the Ras/PI3K/AKT pathway that is known to be important in B cell survival [15,16]. The generation of PI3K by LMP2A also activates mTOR [17] that can further regulate proliferation, growth, differentiation, migration, and survival [17,18,19]. Finally, our laboratory has demonstrated that PI3K signaling induced by LMP2A can promote STAT3 activation, that also contributes to cellular survival [20]. Since mTOR and STAT3 are constitutively active in numerous human cancers, pharmacological inhibition of these pathways are a very active area of clinical research and may be especially important in the treatment of EBV-associated lymphomas.

It has long been understood that cancer cells must rewire the normal cellular mechanisms of ATP production in order to satisfy the increased energy demands for growth and proliferation [21]. While many factors can contribute to meeting this need, one important factor required for the increased production of ATP in tumors is mTOR [22]. The mechanisms by which mTOR increases ATP generation are multifactorial. For example, mTOR elevates protein synthesis through direct phosphorylation of the translational regulators 4E-binding protein 1 (4E-BP1) and p70 ribosomal S6 Kinase (p70 S6K) [23]. The phosphorylation of 4E-BP1 by mTOR at multiple sites releases the eukaryotic initiation factor (eIF4E) which facilitates the selective translation of many important growth factors, such as HIF-1α [24]. However, a more recent study demonstrated that mTOR increased the level of HIF-1α in a translation-independent manner [25]. In this study, the mTOR-dependent increases in HIF-1α were dependent on increases in HIF-1α RNA levels and required the activity of the transcription factor STAT3 [25]. These data suggest that an mTOR/STAT3-dependent axis exists for increasing HIF-1α and HIF-1α-dependent ATP generation. The results of this research are extremely relevant in light of previous data that demonstrate that LMP2A activates both mTOR [17] and STAT3 [20].

In the current study, we ask whether LMP2A alters B cell lymphoma metabolism by increasing ATP generation and the mechanism by which LMP2A achieves these alterations. Our data indicate that LMP2A increases ATP generation through a mTOR/4E-BP1/p70 S6K/HIF-1α-dependent pathway, that does not require STAT3 and changes in HIF-1α RNA levels. These findings implicate the importance of LMP2A in promoting B cell lymphoma survival in tumor environments by increasing ATP generation.

## 2. Results

**Figure 1:**
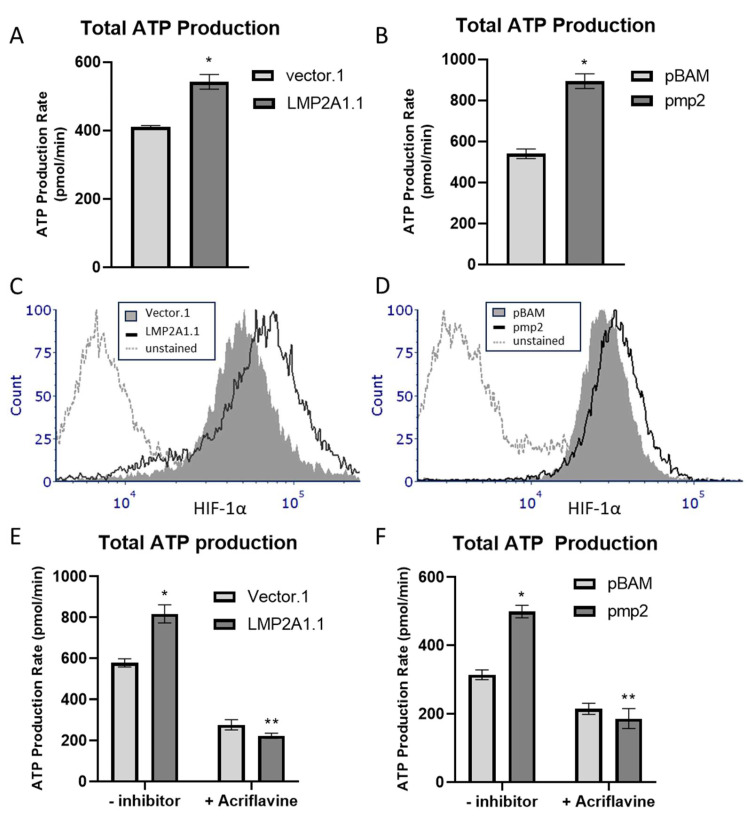
LMP2A increases total ATP production in B cell lymphoma cells by increasing HIF-1α. (**A**,**B**) Stably-transfected LMP2A-negative BJAB (Vector.1) or Ramos (pBAM) and LMP2A-positive BJAB (LMP2A1.1) and Ramos (pmp2) were analyzed using an Agilent Seahorse for total ATP production. (**C**,**D**) LMP2A-negative and -positive cell lines were permeabilized and stained for intracellular HIF-1α as described in the Material and Methods and analyzed using flow cytometry. (**E**,**F**) LMP2A-negative and positive cell lines were incubated in the presence of the HIF-1α inhibitor Acriflavine for 24 h and ATP levels were determined as described in (**A**,**B**). Data are representative of 3–4 experiments. Statistics were performed using Graphpad with a one-way ANOVA followed by a Bonferroni post-hoc test. * indicates *p* < 0.05 when compared to LMP2A-negative cells, ** indicates *p* < 0.05 when compared to LMP2A-positive cells that are not incubated with inhibitor.

B cell receptor (BCR) signaling induces an increase in the amount of ATP available for energy-dependent processes associated with B cell activation [26]. Since LMP2A induces similar upstream pathways as a BCR [11], we hypothesized that LMP2A would increase the generation of ATP. To test this hypothesis, we used the Seahorse XF ATP Production Assay to assess the amount of total ATP generated in LMP2A-negative and -positive B lymphoma cell lines (BJAB and Ramos). As shown in Figure 1A,B, total ATP production was higher in both the BJAB cells (LMP2A.1) and Ramos cells (pmp2) expressing LMP2A in comparison to the cell lines containing only the vector alone (Vector.1 and pBAM) respectively. These data indicate that LMP2A increases ATP generation; however, the mechanism responsible for this increase was unknown.

HIF-1α promotes the generation of ATP in numerous tumor cell types [24,27,28]. We therefore tested whether LMP2A increased HIF-1α levels in B lymphoma cells and if inhibition of HIF-1α blocked the LMP2A-mediated increase in ATP. As shown in Figure 1C,D, when the levels of HIF-1α are assessed by flow cytometry, there is an increase in HIF-1α levels in both BJAB (Figure 1C) and Ramos (Figure 1D) cells that are expressing LMP2A. When an inhibitor of HIF-1α (Acriflavine) is added to the cultures and ATP generation is assessed, the LMP2A-mediated increase in total ATP production is lost (Figure 1E,F), suggesting that LMP2A requires the activity of HIF-1α to increase ATP generation.

**Figure 2:**
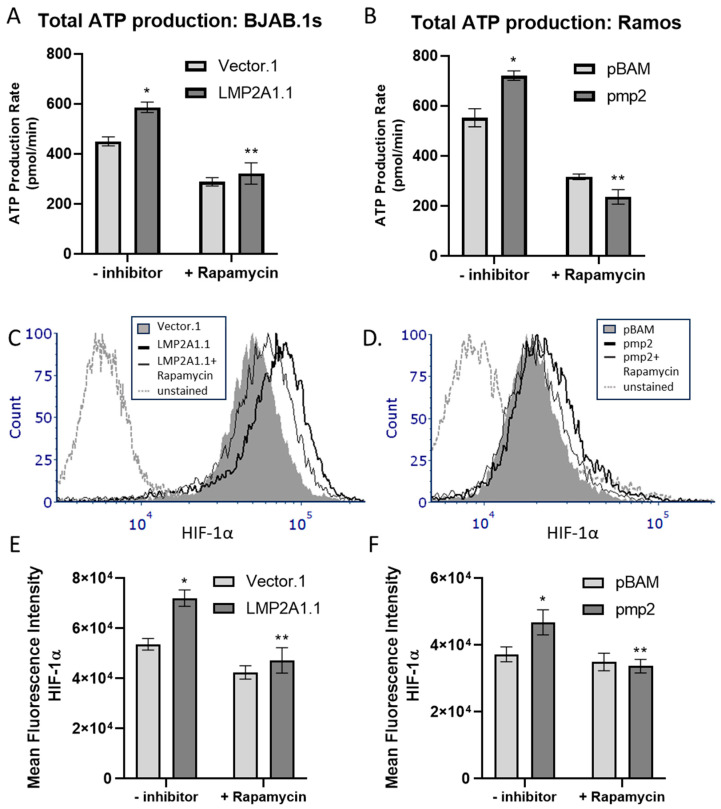
LMP2A activates mTOR to increase total ATP production and HIF-1α levels. (**A**,**B**) LMP2A-negative and positive cells were incubated in the presence of the mTOR inhibitor Rapamycin for 24 h and ATP levels were determined as described in Figure 1A,B. (**C**,**D**) HIF-1α levels were assessed as described in Figure 1C,D after a 24 h incubation in the absence or presence of Rapamycin. (**E**,**F**) Statistical analysis of the increase in HIF-1α in the absence/presence of Rapamycin as determined by flow cytometry. Data are representative of 3–4 experiments. Statistics were performed using Graphpad with a one-way ANOVA followed by a Bonferroni post-hoc test. * indicates *p* < 0.05 when compared to LMP2A-negative cells, ** indicates *p* < 0.05 when compared to LMP2A-positive cells that are not incubated with inhibitor.

Increased activity of mTOR is associated with increases in HIF-1α levels [24,25]. Since LMP2A activates mTOR [17], we tested whether the LMP2A-dependent increase in ATP was mTOR-dependent. To address this question, we incubated LMP2A-negative and -positive BJAB (Figure 2A) or Ramos (Figure 2B) cells with the mTOR inhibitor, rapamycin, for 24 h and we assessed ATP levels using the Agilent Seahorse. As shown in Figure 2A,B, the addition of rapamycin blocked the LMP2A-mediated increase in ATP. Since the ability of LMP2A to increase ATP was dependent on HIF-1α, we tested whether the addition of rapamycin would also decrease HIF-1α levels. As shown in Figure 2C,D, the addition of rapamycin to LMP2A-expressing cells blocked the LMP2A-dependent increase in HIF-1α. Further statistical analysis demonstrates that these changes in HIF-1α are statistically significant (Figure 2E,F).

**Figure 3:**
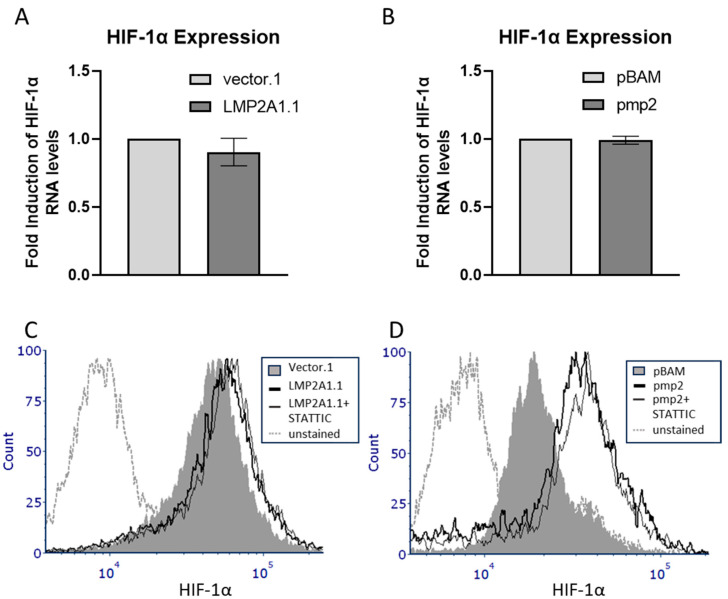
LMP2A does not increase HIF-1α RNA levels and enhanced HIF-1α protein levels are not STAT3-dependent (**A**,**B**) RNA from LMP2A-negative and -positive cell lines were isolated and analyzed for HIF-1α RNA levels by quantitative PCR as described in the Material and Methods. (**C**,**D**) HIF-1α levels were assessed as described in Figure 1C,D in the presence of the STAT3 inhibitor, STATTIC, for 24 h. Data are representative of three experiments.

The ability of mTOR to increase HIF-1α can occur through both transcriptional [25] and post-transcriptional mechanisms [24]. Increases in HIF-1α due to increased transcription require the activity of m-TOR-mediated phosphorylation of STAT3 [29]. We have previously demonstrated that LMP2A increases the tyrosine phosphorylation of STAT3 [20], so we tested whether LMP2A increased HIF-1α at the RNA level and if STAT3 was required to increase HIF-1α. Therefore, we isolated total RNA from LMP2A-negative and -positive cells and analyzed HIF-1α levels by quantitative real-time RT-PCR. As shown in Figure 3A,B, LMP2A-expressing BJAB (LMP2A1.1) and LMP2A-expressing Ramos (pmp2) cells do not have higher levels of HIF-1α RNA (Figure 3A,B), suggesting that the mechanism for increasing HIF-1α levels in LMP2A-expressing cells is post-transcriptional. Consistent with the above findings, the addition of the STAT3 inhibitor, STATTIC, to LMP2A-expressing cells did not influence HIF-1α protein levels in LMP2A-expressing cells as determined by flow cytometry (Figure 3C,D). Finally, under normoxic conditions, protein levels of HIF-1α are regulated by oxygen-dependent hydroxylation and targeting for ubiquitin-mediated degradation by Von Hippel-Lindau (VHL) [30]. If LMP2A increases HIF-1α by decreasing VHL levels, then we would expect to see decreases in LMP2A-expressing cells. To address this, we isolated LMP2A-negative and -positive B cell lines and identified that LMP2A does not decrease VHL expression as determined by flow cytometry (Supplemental Figure S1). Taken together, these data suggest that the LMP2A-dependent increase in HIF-1α requires mTOR activation but does not decrease VHL or utilize STAT3 to increase RNA levels of HIF-1α downstream of mTOR.

**Figure 4:**
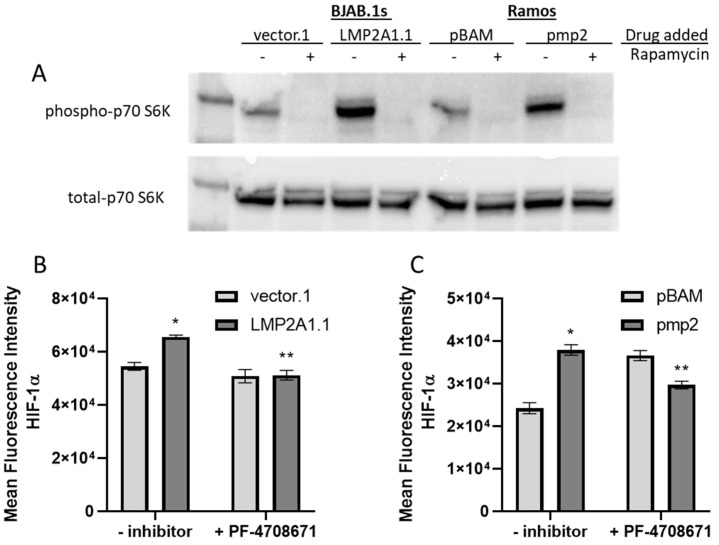
LMP2A uses mTOR to increase p70 S6K activity to increase HIF-1α levels (**A**) Western blot analysis of p70 S6K in LMP2A-negative and -positive cells (**B,C**) HIF-1α levels were assessed as described in Figure 1C,D in the presence of the p70 S6K inhibitor, PF-4708671, for 24 h. Data are representative of 3–4 experiments. Statistics were performed using Graphpad with a one-way ANOVA followed by a Bonferroni post-hoc test. * indicates *p* < 0.05 when compared to LMP2A-negative cells, ** indicates *p* < 0.05 when compared to LMP2A-positive cells that are not incubated with inhibitor.

Alternatively, mTOR elevates protein synthesis by phosphorylating p70 ribosomal S6 Kinase (p70 S6K) [31,32] and 4E-BP1 to release eIF4E [33] that results in increased HIF-1α levels [24]. If LMP2A activates mTOR to increase p70 S6K to increase HIF-1α production, then we would expect higher levels of phospho-p70 S6K in LMP2A-expressing cells that are blocked by the addition of rapamycin. To test this possibility, LMP2A-negative and -positive BJAB and Ramos cell lines were incubated in the absence or presence of rapamycin for 24 h and analyzed for both phospho-p70 S6K and total p70 S6K levels by Western blot analysis. When the levels of phospho-p70 S6K were analyzed, LMP2A-expressing cell lines contained significantly more phospho-p70 S6K than was absent in the presence of rapamycin (Figure 4A). We then tested if phospho-p70 S6K was required for the increase in HIF-1α levels in LMP2A-expressing cell lines. When the PF-4708671 inhibitor was added to block p70 S6K, the levels of HIF-1α were decreased in comparison to LMP2A-expressing cell lines incubated in the absence of an inhibitor (Figure 4B,C), suggesting that p70 S6K contributes to the LMP2A-mediated increase in HIF-1α.

**Figure 5:**
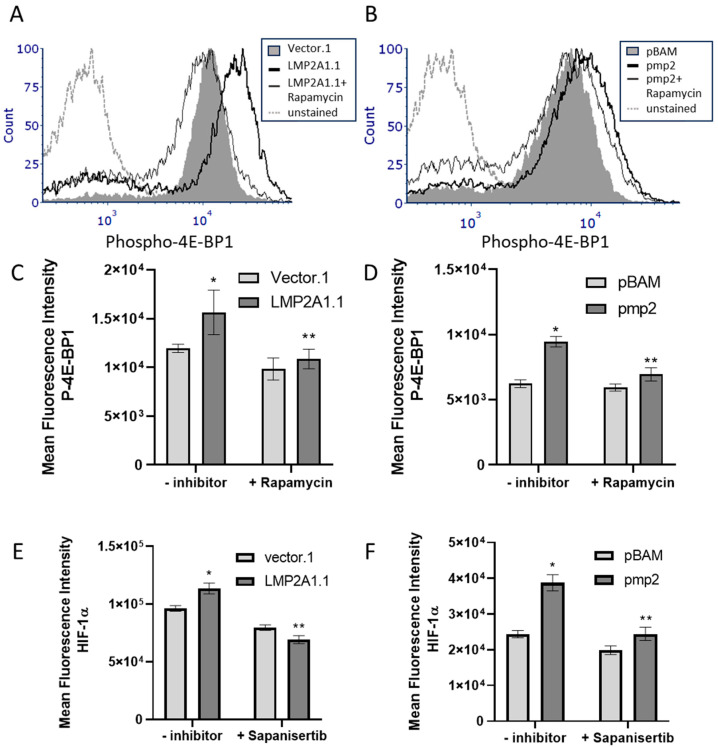
LMP2A uses mTOR to increase phosphorylation of 4E-BP1 to increase HIF-1α levels (**A**,**B**) LMP2A-negative and -positive cell lines were permeabilized and stained for intracellular phospho-4E-BP1 levels as described in the Material and Methods. (**C**,**D**) Statistical analysis of the increase in phospho-4E-BP1 in the absence/presence of Rapamycin as determined by flow cytometry. (**E**,**F**) HIF-1α levels were assessed as described in Figure 1C,D in the presence of the 4E-BP1 inhibitor, Sapanisertib, for 24 h. Data are representative of 3–4 experiments. Statistics were performed using Graphpad with a one-way ANOVA followed by a Bonferroni post-hoc test. * indicates *p* < 0.05 when compared to LMP2A-negative cells, ** indicates *p* < 0.05 when compared to LMP2A-positive cells that are not incubated with inhibitor.

HIF-1α levels can also be increased through the mTOR-dependent phosphorylation of 4E-BP1 [24]. Therefore, we tested whether LMP2A increased the levels of phosphorylated 4E-BP1 in BJAB and Ramos cell lines in an mTOR-dependent manner. As shown in Figure 5A,B, our data indicate that LMP2A-expressing cells have significantly higher levels of phospho-4E-BP1, which was lost in the presence of rapamycin. Further statistical analysis demonstrates that these changes in phospho-4E-BP1 are statistically significant (Figure 5C,D). To determine if LMP2A requires phospho-4E-BP1 to increase HIF-1α levels, we added Sapanisertib to block the generation of phospho-4E-BP1 in LMP2A-negative and -positive cell lines. When Sapaniesertib was added to LMP2A-expressing cell lines, the levels of HIF-1α were decreased in comparison to LMP2A-expressing cell lines with no inhibitor (Figure 5E,F). These data indicate that the LMP2A-mediated activation of mTOR promotes both p70 S6K and phospho-4E-BP1 activation to increase HIF-1α levels that are required for LMP2A to increase ATP generation.

**Figure 6:**
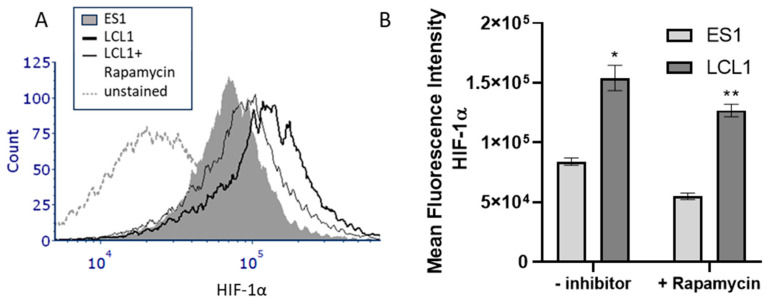
LMP2A increases HIF-1α levels in the context of EBV latency proteins. (**A**) LMP2A-negative (ES1) and -positive (LCL1) lymphoblastoid cell lines were permeabilized and stained for intracellular HIF-1α levels as described in the Material and Methods. (**B**) Statistical analysis of the increase in HIF-1α in the absence/presence of rapamycin as determined by flow cytometry. Data are representative of 3–4 experiments. Statistics were performed using GraphPad with a one-way ANOVA followed by a Bonferronic post-hoc test. * indicates *p* < 0.05 when compared to LMP2A-negative cells, ** indicates *p* < 0.05 when compared to LMP2A-positive cells that are not incubated with inhibitor.

The data thus far utilized two unique Burkitt’s lymphoma cell lines that are stably transfected either with vector alone or vector with LMP2A. To assess whether LMP2A would increase HIF-1α in the context of other latency proteins, we analyzed LMP2A-negative (ES1) or LMP2A-positive (LCL1) lymphoblastic cell lines that express all latency proteins [34] for HIF-1α levels by flow cytometry. As shown in Figure 6A, LMP2A-positive LCL1 cells express significantly more HIF-1α than the LMP2A-negative ES1 lymphoblastoic cell line. Since multiple EBV latency proteins can influence HIF-1α expression [35], we tested whether the enhancement in HIF-1α in LMP2A-expressing lymphoblastoid cell lines was mTOR dependent. As shown in Figure 6A,B, the data demonstrate that the addition of rapamycin to LMP2A-expressing LCL1 cells significantly decreases HIF-1α levels in comparison to LCL1 cells incubated in the absence of rapamycin. Additionally, we confirmed that LMP2A did not increase VHL levels in these cells (Supplemental Figure S1). Taken together, these data confirm that LMP2A increases HIF-1α levels in the presence of the entire virus and other EBV latency proteins.

## 3. Discussion

EBV was the first oncogenic virus identified over 60 years ago [36]. Since that time, the relationship of EBV to the transformation and survival of lymphomas has been intensely studied. EBV is associated with the development of Burkitt’s lymphoma, classic Hodgkin’s lymphoma, and diffuse large B cell lymphoma. While EBV is not required for the development of these lymphomas, EBV-associated lymphomas tend to be more resistant to chemotherapy [34]. Data from numerous laboratories indicate that almost all EBV latency genes have some capacity to contribute to increased survival and transformation of B cells, but the consistent presence of LMP2A in all forms of EBV-associated lymphomas [34] indicates an important function of this protein not only for tumor development and survival but also in regards to treatment efficacy. Our current data are the first to identify a new function for LMP2A as a positive regulator of HIF-1α and ATP production in B cell lymphomas through a post-transcriptional mechanism that is dependent on p70S6K and 4E-BP1. Due to the emerging role and importance of HIF-1α in promoting chemotherapeutic resistance [37], our data suggest that the LMP2A-mediated increase in HIF-1α may contribute to EBV-mediated evasion of chemotherapy.

Quiescent, non-proliferating cells have different metabolic needs than cells that are being activated [38] or in this case, cells, expressing viral proteins [39]. Viruses need to control metabolism at multiple time points in their life cycle. Early on, during active viral replication, viruses need to promote the replication of their genome as well as induce viral protein production, which requires the generation of not only energy but metabolic by-products that serve as a source of amino acids and nucleotides [39]. For many oncogenic viruses, oncogenic transformation occurs years later and via numerous mechanisms. However, one requirement for all of these processes is the need to modify metabolism so that uncontrolled proliferation and survival can occur [40]. While viruses have developed many pathways to alter metabolisms, almost all oncogenic viruses increase HIF-1α levels via multiple pathways [41].

As pointed out by Yang et al., numerous EBV-encoded oncogenes have been analyzed for their impact on metabolic enzymes, including HIF-1α [35]. EBV increases ATP generation through multiple pathways, indicating that it is in the virus’s best interest to enhance the availability of ATP for survival and/or re-entry into the lytic pathway [35,42,43]. During EBV latency, the virus exists in multiple latency states [8,44]. In proliferating, newly-infected naïve B cells and EBV-associated post-transplant lymphoproliferative B cell disorders, EBV expresses Stage III latency in which all latency proteins (all EBNAs, LMP1, LMP2A, LMP2B) and non-encoding EBV-encoded RNAs (EBERs) and microRNAs (miRNAs) are expressed. During Stage II latency, which is found in germinal center B cells and EBV-associated lymphomas, there is more restriction on gene expression in which EBNA-1, LMP1, LMP2A, EBERs, and miRNAs are produced. In the Latency Zero and Latency I stages, which are found in memory B cells, there is even more restriction on EBV gene expression in which only EBNA1, EBERs, and miRNAs are expressed in order to hide from the immune system [8,44]. Of these proteins listed above, EBV utilizes EBV latency proteins found in each of these latency programs to enhance B cell metabolism and/or HIF-1α production. Initial findings indicated that EBNA1, which is found in all stages of latency can positively regulate HIF-1α transcription in epithelial cell lines [45]. While this has not been demonstrated in B cell lines, it suggests the possibility that EBNA1 can specifically promote HIF-1α production in memory B cells as needed. Additionally, previous data demonstrate that LMP1, which is found in Stages II and III of EBV latency, also enhances HIF-1α in a post-transcriptional pathway that requires a key E3 ubiquitin ligase that induces the degradation of prolyl hydroxylases (PHDs) required for HIF-1α breakdown [46]. Additionally, the latency proteins EBNA3A and EBNA-LP that are found in Stages II and III of latency bind and block the activity of PHDs to block the oxygen-dependent degradation of HIF-1α [47]. While EBV latency proteins from all stages of EBV latency can promote the expression and/or stabilization of HIF-1α, our data identify LMP2A as a source of a unique pathway that increases the activation of pro-translational factors to increase HIF-1α that are independent of modulating HIF-1α transcription or degradation.

Furthermore, HIF-1α is targeted by many oncogenic viruses in both hypoxic and oxygen-rich environments [41]. Our studies were performed under standard conditions in which oxygen is not limiting. Consistent with the literature, previous studies have indicated the importance of mTOR in ATP generation when oxygen is not limiting [48,49]. However, it would be interesting to determine if LMP2A also promotes mTOR-dependent increases in ATP under hypoxic or anoxic conditions. That being said, previous studies using cell lines that were exposed to hypoxic conditions induced a switch from latency to lytic replication [50,51] and required the involvement of HIF-1α [50]. However, in some of these experiments, HIF-1α was overexpressed and the normal regulation of HIF-1α was lost, which may impact the interpretation of these data. Nevertheless, the idea of regulating HIF-1α from the EBV’s point of view makes sense. To this point, if an EBV-infected cell is starved for oxygen, then it is advantageous for the virus to “jump ship” and engage in the lytic cycle. However, under non-hypoxic conditions, for example, during the onset of tumorigenesis, moderate increases of HIF-1α can promote the increased generation of ATP to meet the increased demands for energy to replicate and promote survival. Future studies that address the ability of LMP2A to increase ATP generation under hypoxic conditions would be interesting to mimic later stages of tumor development, in which oxygen levels become limiting in the solid tumor microenvironment.

LMP2A has historically been considered purely a BCR mimic due to the activation of Lyn and Syk through its ITAM motifs [52,53] that are also found in the signaling molecules Igα and Igβ associated with surface immunoglobulin [54]. However, both historical and more recent data indicate that while there are some similarities between LMP2A and BCR signaling, downstream LMP2A signaling is unique in regards to the molecules that are constitutively activated in resting and transformed cells [9,11,55]. Recent genomic studies that analyzed the phosphorylation of tyrosine residues between LCLs that were stimulated through the BCR or only through LMP2A demonstrated similarities in upstream activated proteins (such as Lyn, SYK, BTK) but only 12% in similarities further downstream [11]. These differences were hypothesized to promote cellular survival and proliferation in EBV-infected cells, which is the perfect situation to promote tumor development. When one combines the previously known impact on cellular signaling with the ability to increase ATP generation, EBV ultimately can promote not only tumor development but also provide increased energy to meet the metabolic needs of the emerging tumor [42].

In Burkitt’s lymphoma, EBV-mediated activation of the mTOR pathway correlated with fewer mutations in developed cancers, which the authors suggested was due to the presence of activated mTOR that bypasses the need for oncogenic mutations [56]. Interestingly, previous studies using a mouse model of Burkitt’s lymphoma that expressed LMP2A bypasses the need for mutations of the oncogene p53 [57]. While this paper did not identify the signals from LMP2A that are required to reach this effect, subsequent studies using this same mouse model of Burkitt’s lymphoma demonstrated that the mTOR inhibitor, rapamycin, blocks the LMP2A-mediated enhancement in tumor development [58]. Our current information suggests that an mTOR-dependent enhancement of ATP production and HIF-1α levels may contribute to this LMP2A-dependent enhancement in MYC-driven tumor development and potentially the need to bypass additional oncogenic mutations. One limitation of our current work is that we are only analyzing LMP2A function in fully developed B cell lymphoma cell lines. Future works that determine if LMP2A activates mTOR/ p70 S6K/4E-BP1 to enhance HIF-1α and ATP production in pre-malignant cells would be important to understand the contribution of this pathway to tumor development in EBV-associated tumors. Additionally, while we have addressed the ability of LMP2A to function in the context of the entire virus (Figure 6), since LMP1 and LMP2A activate similar pathways (PI3K, NF-kB, STAT3) [20,59,60,61] and have been shown to cooperate in promoting B cell lymphoma development [62], future studies should analyze if LMP1 and LMP2A enhance HIF-1α and ATP generation in an additive or synergistic manner.

Finally, a previous study indicated that EBV uses mTOR-dependent pathways to increase metabolic activity in Burkitt’s lymphoma cell lines [63]. Our data indicate that the LMP2A-mediated activation of mTOR and ultimately ATP generation could contribute to these findings. These findings are important in that multiple types of tumors that overexpress activated mTOR are more resistant to chemotherapy [64]. Furthermore, inhibitors of mTOR were less successful in clinical studies in the treatment of B cell lymphomas than expected based on pre-clinical experiments [21], suggesting the need for multi-target approaches that include inhibitors of mTOR in conjunction with HIF-1α. For EBV-associated tumors, analysis of LMP2A, mTOR activation, and/or HIF-1α levels may allow for optimized chemotherapeutic approaches that target multiple pathways.

## 4. Materials and Methods

### 4.1. Cell Lines

Human Burkitt’s B cell lymphoma cell lines (BJAB and Ramos), which were generously provided by Dr. Richard Longnecker (Northwestern University), express LMP2A (LMP2A1.1 and pmp2) or vector alone (vector.1 and pBAM), while EBV-transformed LCL cell lines either express LMP2A (LCL1) or are deficient of LMP2A (ES1) as described and published previously [59,65]. Briefly, the BJAB cell lines were stably transfected using electroporation with a vector that provided for selection with gentamycin and hygromycin and included a gene encoding LMP2A (LMP2A1.1) or the vector alone (Vector.1) [59]. The Ramos cell lines were generated by retroviral transduction with 4 μg/mL polybrene and supernatants containing either the pBAMHYGRO (pBAM) vector retrovirus or the PMP2LMP2A (pmp2) retrovirus encoding for LMP2A for 24 h followed by selection with hygromycin [65]. Cells were grown in complete Roswell Park Memorial Institute (RPMI) 1640 media with L-glutamine, 10% fetal calf serum, and 1% penicillin/streptomycin and were maintained at 37 °C, 5% CO_2_. Stable retention of the vector DNA or vector + LMP2A DNA in BJAB or Ramos cell lines was maintained by growth in cRPMI + 2 μg/mL gentamycin + 400 μg/mL hygromycin (BJAB) or only 400 μg/mL of hygromycin (Ramos) as described previously [59,65]. Inclusion criteria included using equal numbers of viable cells for analysis and that cells be used within 2–8 passages after thawing. LMP2A expression in all LMP2A-positive cell lines was confirmed by immunofluorescence (Supplemental Figure S2).

### 4.2. Seahorse XF ATP Production Assay

BJAB or Ramos cells were plated in 6-well plates in complete RPMI at a concentration of 2 × 10^6^ cells per well, either in the absence or presence of Rapamycin (1 µM) (Sigma-Aldrich, St. Louis, MO, USA) or Acrivlafine (5 µM) (MedChemExpress, Monmouth Junction, NJ, USA). Additionally, a 24-well plate was coated overnight using 0.1mg/mL Poly-D-Lysine (Thermo Fisher Scientific, Waltham, MA, USA) to adhere suspension cells to the bottom of the plate. After 24 h at 37 °C, 5% CO_2_, cells were plated in complete RPMI at a concentration of 7.5 × 10^4^ cells per well, at a minimum in triplicate, into a 24-well plate. After a 2 h incubation at 37 °C, 5% CO_2_, media was replaced with phenol-free RPMI (Agilent, Santa Clara, CA, USA) and incubated for 1 h in a CO_2_-free 37 °C incubator. During incubation, oligomycin and rotenone/antimycin A compounds were pipetted into specific injection ports on an XF24 Sensor Cartridge (Agilent), after being hydrated overnight using XF Calibrant (Agilent) in a CO_2_-free 37 °C incubator. After loading of compounds, the Sensor Cartridge was loaded into the Seahorse XFe24 Analyzer (Agilent) and following calibration, the 24-well plate containing cells was loaded into the analyzer. Serial injections of oligomycin, followed by rotenone/antimycin A into the 24-well plate were performed to determine the kinetic profile of Oxygen Consumption Rate (OCR) and Extracellular Acidification Rate (ECAR) measurements. These data were exported to the XF Real-Time ATP Rate Assay Report Generator, which was used to calculate the ATP production rate.

### 4.3. Flow Cytometry

BJAB or Ramos cell lines were plated at a concentration of 1 × 10^6^ cells per well in triplicate using 12 well plates in complete RPMI for 24 hrs at 37 °C, 5% CO_2_ in the absence or presence of either Rapamycin (1 µM) (Sigma-Aldrich), STATTIC (1.75 μM) (Sigma-Aldrich), PF-4708671 (20 µM) (Selleckchem, Houston, TX, USA) or Sapanisertib (0.1 µM) (Selleckchem). For a flow chart of analysis of all samples, please see Supplemental Figure S3. After incubation, cells were washed using PBS by centrifuging at 1500 rpm for 5 min, followed by incubation with Zombie Violet™ Viability dye (Biolegend, San Diego, CA, USA). After PBS washes, cells were then blocked using TruStain FcX™ Fc Receptor Blocking Solution (Biolegend) for 30 min at 4 °C. Cells were then fixed and permeabilized using eBioscience™ IC Fixation Buffer and Permeabilization Buffer as recommended by the vendor (Thermo Fisher Scientific). Following subsequent washes, cells were stained with PE-conjugated HIF-1α antibody (Biolegend), anti-4E-BP1 (Cell Signaling Technologies, Danvers, MA), or anti-Von Hippel Lindau (VHL) antibody (Thermo Fisher Scientific). Samples were run using the CytoFLEX Flow Cytometer (Beckman Coulter, Indianapolis, IN), and flow cytometry data were analyzed using FCS Express 6 Plus software (De Novo Software, Pasadena, CA, USA). Quality control (QC) was performed daily prior to use and included adherence to specific criteria based against a specific lot of QC Fluorospheres (Beckman Coulter, Indianapolis, IN, USA) including a gain difference of less than or equal to 20% from the target gain, the median fluorescence intensity (MFI) difference less than or equal to 5% from the target MFI, as well as a robust coefficient of variation (rCV) value less than or equal to 5%. Experiments were only run on CytoFLEX after the QC run was successful.

### 4.4. RT-PCR

BJAB or Ramos cell lines were plated at a concentration of 2 × 10^6^ cells per well using 6 well plates in complete RPMI at 37 °C, 5% CO_2_. After 24 h, RNA was isolated using E.Z.N.A.^®^ HP Total RNA Kit (OMEGA bio-tek, Nocross, GA, USA) according to the manufacturer’s instructions. Then, RNA was quantified using NanoDrop™ 2000 Spectrophotometer (Thermo Fisher Scientific). Equal amounts of RNA were reverse transcribed into cDNA using qScript cDNA SuperMix (Quantabio, Beverly, MA, USA) and cDNA was loaded to wells of a 96-well plate along with TaqMan^®^ probe and primers and TaqMan^®^ Advanced Master Mix (Thermo Fisher Scientific). *HIF-1α* gene expression along with GAPDH were quantified and analyzed using the QuantStudio™ 5 Real-Time PCR machine (Thermo Fisher Scientific). GAPDH was used as an endogenous control for each experimental group in order to calculate the relative gene expression of samples. A minimum of three replicates were used per experimental group with variation in CT values among replicates less than 0.5. Samples containing nuclease-free water in place of experimental groups were also included to check for contamination or non-specific amplification. Fold induction was determined by comparing changes in ΔΔC_t_ values to control levels. The QuantStudio™ 5 RT-PCR System undergoes maintenance and calibration by a technical specialist as recommended by the manufacturer.

### 4.5. Western Blot

BJAB or Ramos cell lines were plated at a concentration of 2 × 10^6^ cells per well in the absence or presence of rapamycin (1 μM) using 6 well plates in complete RPMI at 37 °C, 5% CO_2_. After 24 h, protein lysates were generated by first lysing cells using Ripa Lysis Buffer containing Halt™ Protease and Phosphatase Inhibitors (Thermo Fisher Scientific). Protein lysates were quantified using Pierce™ BCA Protein Assay (Thermo Fisher Scientific) and equal amounts of protein were then prepared with Novex™ Tris-Glycine SDS Sample Buffer (Thermo Fisher Scientific) prior to being loaded into Bolt™ Bis-Tris Plus Mini Protein Gels and running at 200 volts for 20 min. Gels were then transferred onto PVDF membranes (Thermo Fisher Scientific) for one hour at 20 volts. Membranes were then blocked for one hour using 1% casein in tris-buffered saline (TBS), followed by overnight incubation at 4 °C with (1:1000) anti-phospho-p70 S6 Kinase (Thr389) or anti-p70 S6 Kinase (Cell Signaling Technologies) antibodies. The membranes were then washed using TBS with 0.1% Tween-20 (TBS-T) and incubated using (1:5000) goat anti-rabbit IgG secondary antibody, HRP (Thermo Fisher Scientific). Following subsequent TBS-T washes, membranes were incubated with ECL detection reagent (GE Healthcare, Chicago, IL, USA) prior to imaging chemiluminescent signals using the ChemiDoc™ MP Imaging System (Bio-Rad Laboratories, Hercules, CA, USA).

### 4.6. Statistics

For all metabolism and flow cytometry experiments, each cell line was plated at minimum in triplicate in the absence or presence of an inhibitor for 24 h. At the end of this time period, each well was individually analyzed using either the Agilent Seahorse (ATP production) or CytoFLEX Flow cytometer. For the RT-PCR experiments, cells were plated and RNA isolation after 24 h was performed for each experimental group. Normalized RNA amounts were then reverse transcribed to cDNA and qPCR for each sample was performed in triplicate to ensure accuracy of the results using the Quantstudio™ 5 Real-time PCR machine. Each determined value was averaged for each experimental group and then the experiments were analyzed using GraphPad Prism 7 Software (SanDiego, CA, USA). For all experiments, statistical significance was determined by performing a two-way analysis of variance followed by a Bonferroni post-hoc test. A *p*-value of <0.05 was considered statistically significant. Each of these individual experiments described above was performed at least three times independently to demonstrate reproducibility.

## 5. Conclusions

In contrast to other EBV latency proteins that enhance HIF-1α levels through transcriptional or degradation-dependent pathways, the data presented herein demonstrate a novel mechanism in which EBV LMP2A directly promotes the increase in pro-translational factors (p70 S6K and 4-EB-P1) to increase HIF-1α and ATP production in an mTOR-dependent pathway. The constitutive activation of mTOR by LMP2A and enhanced ATP production may contribute to chemotherapeutic resistance of EBV-related lymphomas. In a medical age that is moving towards precision medicine, findings such as these suggest that establishing EBV association with B cell lymphomas may be needed to optimize treatment strategies.

## Data Availability

Data are contained within the article and Supplementary Materials.

## Supplementary Materials

The following supporting information can be downloaded at: https://www.mdpi.com/article/10.3390/ijms25073944/s1.
