# Supplementary material for: Epstein–Barr Virus Latent Membrane Protein 2A (LMP2A) Enhances ATP Production in B Cell Tumors through mTOR and HIF-1α
Source: Int J Mol Sci. 2024 Apr 2;25(7):3944. doi: 10.3390/ijms25073944 (PMC11012313; doi:10.3390/ijms25073944)
Supplement: Supplementary file 1 [file ijms-25-03944-s001.zip › ijms-2898260-supplementary.pdf]

Supplementary Materials

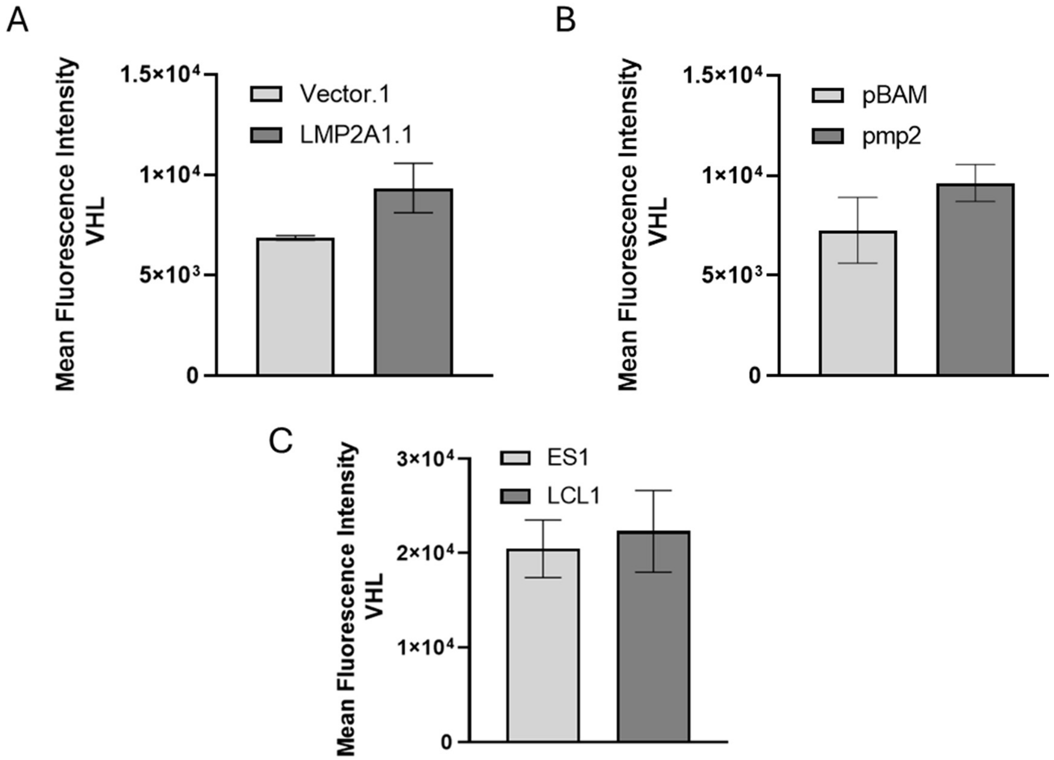

Figure S1. LMP2A does not decrease VHL intracellular protein levels. (A–C) LMP2A-negative and -positive cell lines were permeabilized and stained for intracellular VHL as described in the Material and Methods and analyzed using flow cytometry. Data are representative of 2 experiments with similar results.

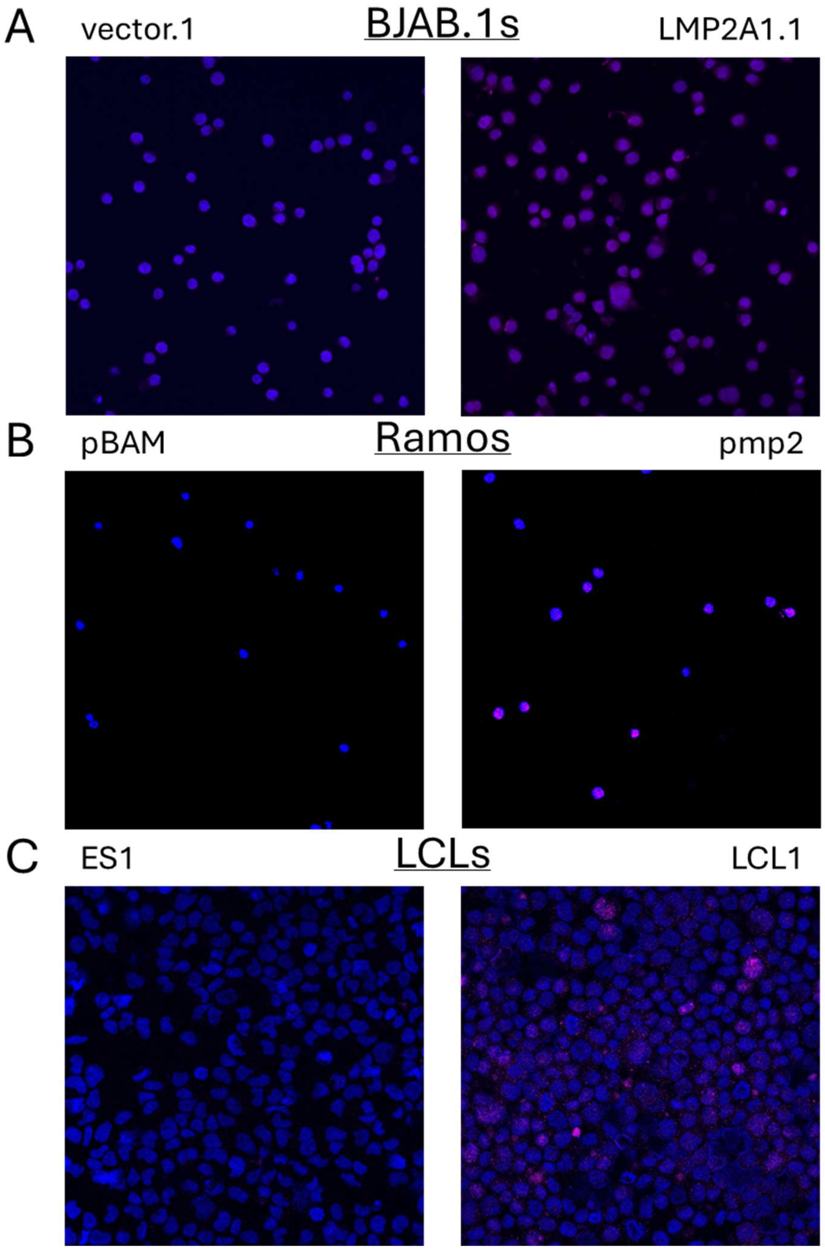

Figure S2. LMP2A expression in B cell lymphoma cell lines. LMP2A-negative (BJAB.1s-Vector.1, Ramos-pBAM) and LMP2A-positive (BJAB.1s-LMP2A1.1, Ramos-pmp2) B cell lines or EBV-positive lymphoblastoid cell lines (LCLs) containing LMP2A (LCL1) or absent of LMP2A (ES1) were fixed using IC Fixation Buffer (Thermo Fisher Scientific). Cells were then permeabilized using 1X Permeabilization Buffer (Thermo Fisher Scientific) and subsequently blocked with 10% goat serum in PBS. Cells were incubated with primary antibody rat anti-LMP2A (1:100, clone 14B7) (Thermo Fisher Scientific) followed by secondary antibody using (**A**,**B**) anti-Rat IgG-Cy3 (Biolegend) or (**C**) anti-Rat IgG-DyLight™ 650 (Thermo Fisher Scientific). Using a cytospin, cells were transferred to slides and attached to coverslips using VECTASHIELD® HardSet™ Mounting Medium containing DAPI (Vector Laboratories). Secondary antibody only stained samples were also analyzed to confirm specificity of primary antibody (data not shown). Images were taken using 60× oil objective on the Nikon A1R laser scanning confocal microscope.

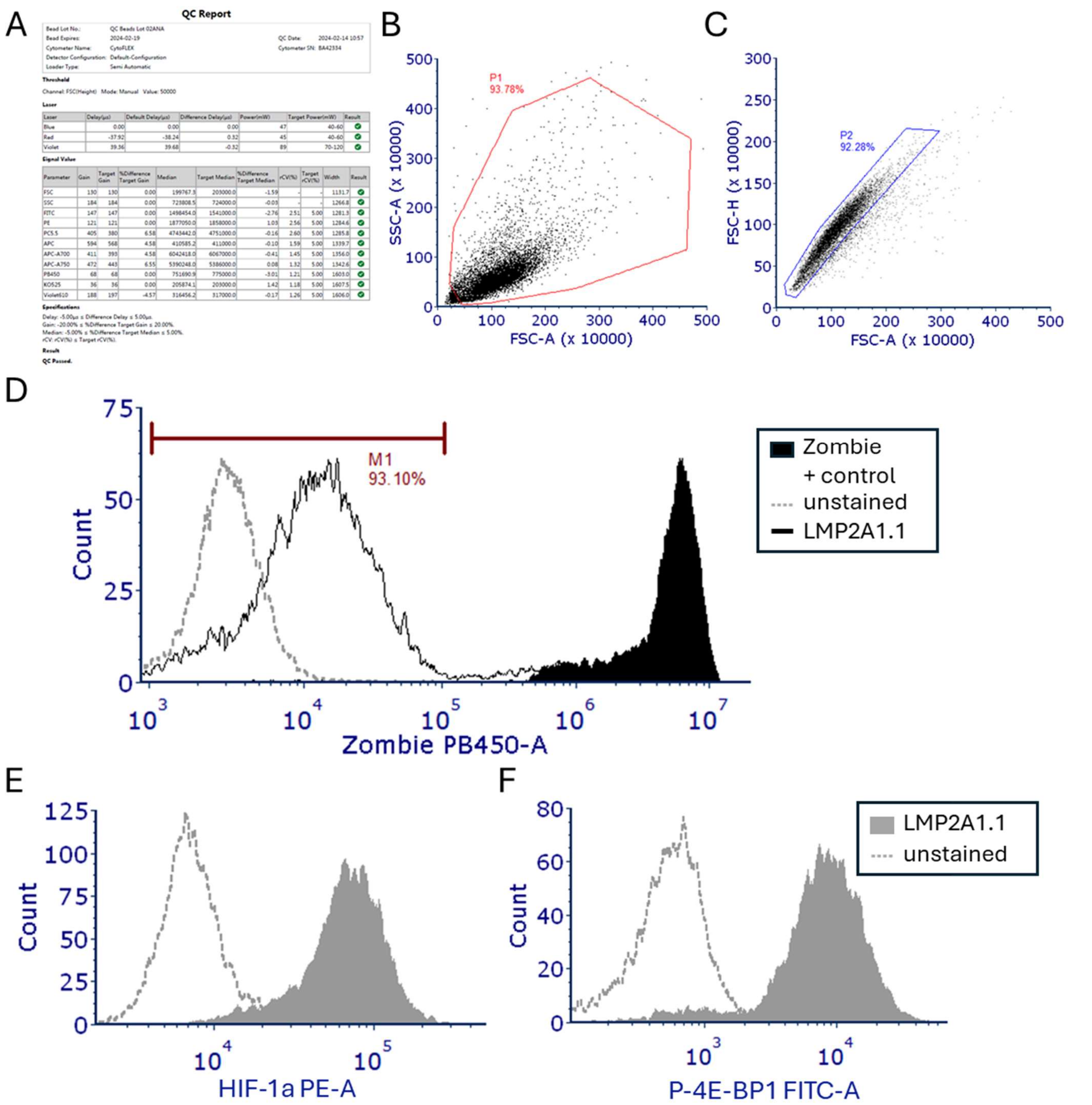

QC Report

Bead Lot No.:	QC Beads Lot 02ANA		
Bead Expires:	2024-02-19	QC Date:	2024-02-14 10:57
Cytometer Name:	CytoFLEX	Cytometer SN:	BA42334
Detector Configuration:	Default-Configuration		
Loader Type:	Semi Automatic		

Threshold

Channel: FSC(Height) Mode: Manual Value: 50000

Laser

Laser	Delay(μs)	Default Delay(μs)	Difference Delay(μs)	Power(mW)	Target Power(mW)	Result
Blue	0.00	0.00	0.00	47	40-60	✓
Red	-37.92	-38.24	0.32	45	40-60	✓
Violet	39.36	39.68	-0.32	89	70-120	✓

Signal Value

Parameter	Gain	Target Gain	%Difference Target Gain	Median	Target Median	%Difference Target Median	rCV(%)	Target rCV(%)	Width	Result
FSC	130	130	0.00	199767.3	203000.0	-1.59	-	-	1131.7	✓
SSC	184	184	0.00	723808.5	724000.0	-0.03	-	-	1266.8	✓
FITC	147	147	0.00	1498454.0	1541000.0	-2.76	2.51	5.00	1281.3	✓
PE	121	121	0.00	1877050.0	1858000.0	1.03	2.56	5.00	1284.6	✓
PC5.5	405	380	6.58	4743442.0	4751000.0	-0.16	2.60	5.00	1285.8	✓
APC	594	568	4.58	410585.2	411000.0	-0.10	1.59	5.00	1339.7	✓
APC-A700	411	393	4.58	6042418.0	6067000.0	-0.41	1.45	5.00	1356.0	✓
APC-A750	472	443	6.55	5390248.0	5386000.0	0.08	1.32	5.00	1342.6	✓
PB450	68	68	0.00	751690.9	775000.0	-3.01	1.21	5.00	1603.0	✓
KO525	36	36	0.00	205874.1	203000.0	1.42	1.18	5.00	1607.5	✓
Violet610	188	197	-4.57	316456.2	317000.0	-0.17	1.26	5.00	1606.0	✓

Specifications

Delay: -5.00μs ≤ Difference Delay ≤ 5.00μs.
Gain: -20.00% ≤ %Difference Target Gain ≤ 20.00%.
Median: -5.00% ≤ %Difference Target Median ≤ 5.00%.
rCV: rCV(%) ≤ Target rCV(%).

Result

QC Passed.

Figure S3. B cell gating strategy to determine mean fluorescent intensity of HIF-1α or phospho-4E-BP1 (**A**) Quality control (QC) calibration using CytoFLEX Daily QC Fluorospheres (Beckman Coulter) was performed prior to running all experiments on the CytoFLEX flow cytometer to ensure accuracy and precision of data. (**B**) Cells were gated (P1) using forward side scatter vs. side scatter plots in order to exclude any debris. (**C**) P1-gated cells were then gated further (P2) using forward scatter area vs. forward side scatter height plots to include single cells and exclude any particles or clumped cells. (**D**) P2 gated cells were gated using histograms measuring fluorescence of Zombie Violet™ Fixability Viability dye (Biolegend) in order to assess live (M1) vs. dead cells. (**E**,**F**) Zombie-negative gated cells (M1) were analyzed using histograms to measure mean fluorescent intensity of HIF-1α-PE (Biolegend) or phospho-4E-BP1-FITC (Cell Signaling Technologies). This strategy was enlisted for every experiment with every sample analyzed according to this strategy.
